# First hemispheric report of invasive tick species *Haemaphysalis punctata*, first state report of *Haemaphysalis longicornis*, and range expansion of native tick species in Rhode Island, USA

**DOI:** 10.1186/s13071-021-04887-z

**Published:** 2021-08-10

**Authors:** Danielle M. Tufts, Maria A. Diuk-Wasser

**Affiliations:** 1 Infectious Diseases and Microbiology Department, University of Pittsburgh, 2119 Public Health, 130 De Soto St, Pittsburgh, PA USA; 2 Ecology, Evolution, and Environmental Biology Department, Columbia University, 1200 Amsterdam Ave, New York, NY USA

**Keywords:** Asian longhorned tick, Red sheep tick, Lone star tick, Rabbit tick, Tick-borne pathogens, Invasive species

## Abstract

**Background:**

Invasive arthropod vectors and the range expansions of native vectors can lead to public and veterinary health concerns, as these vectors may introduce novel pathogens or spread endemic pathogens to new locations. Recent tick invasions and range expansion in the USA has been attributed to climate and land use change, an increase in global travel, and importations of exotic animals.

**Methods:**

A 10-year surveillance study was conducted on Block Island, Rhode Island, from 2010 to 2020 including sampling ticks from small mammal and avian hosts.

**Results:**

We report the discovery and establishment of the red sheep tick (*Haemaphysalis punctata*) for the first time in the western hemisphere and in the US. This invasive species was first collected in 2010 on Block Island, was collected continuously throughout the study, and was collected from an avian host. We document the first report of the invasive Asian longhorned tick (*Haemaphysalis longicornis*) in the state of Rhode Island, first observed at our sites in 2018. Finally, we present data on the range expansion and establishment of two native tick species, the lone star tick and the rabbit tick, on Block Island.

**Conclusion:**

This study emphasized the importance of long-term surveillance to detect changes in tick host communities, including invasive and expanding native vectors of potential significance to humans and wildlife.

**Graphical abstract:**

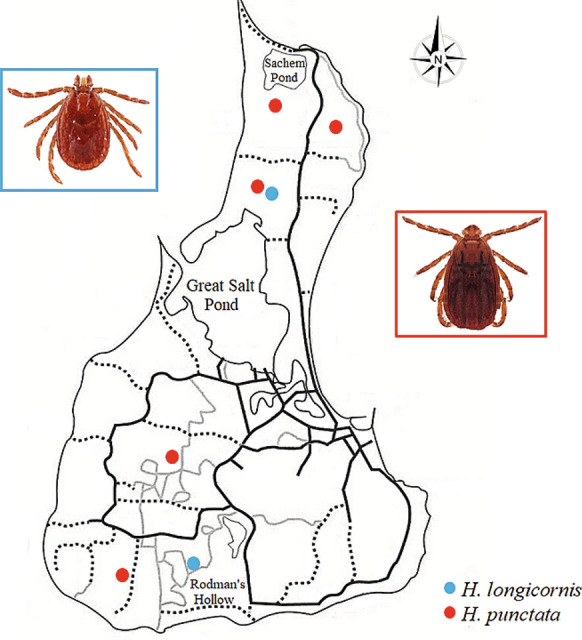

**Supplementary Information:**

The online version contains supplementary material available at 10.1186/s13071-021-04887-z.

## Background

The invasion and establishment of new arthropod vectors may lead to the introduction of new pathogenic threats to wildlife, humans, and domestic animals [1]. New vector invasions and geographic expansion from historical ranges have been attributed to climate and anthropogenic land use changes, geographic distribution and abundance of host species, an increase in global travel, and importations of exotic animals [2, 3]. Vector invasions and range expansions may lead to changes in ecological characteristics including competition with and displacement of native species [4]. Ticks with a generalist feeding behavior may be more likely to expand their ranges when they feed on host species with wide distribution ranges such as migratory birds and large mammals [5]. However, the potential for invasive tick species to transmit novel or native infectious diseases remains relatively unpredictable without laboratory and field experiments.

A recent invasive species in the USA is the Asian longhorned tick, *Haemaphysalis longicornis*, which was first documented from New Jersey in 2017; however, misidentified specimens were reported from West Virginia in 2010 and it has rapidly spread across 15 states in the eastern USA [6, 7]. Additionally, predictive models suggest the range of *H. longicornis* will continue expanding westward [8]. In some regions of its invasive range in the USA, *H. longicornis* has been found to be PCR positive for various pathogens including introduced (e.g. *Theileria orientalis*) and native (e.g. *Borrelia burgdorferi*) species [9, 10]. In laboratory studies, *H. longicornis* were found to be competent vectors of *Rickettsia rickettsii* [11], but were not competent to transmit *B. burgdorferi* [12] or *Anaplasma phagocytophilum* [13]. While humans may not be a preferred host for *H. longicornis* [14], the incidence of this tick species feeding on humans in the USA is increasing [15].

The red sheep tick, *Haemaphysalis punctata*, is native to the Palearctic region (including southern Europe, southwest Asia, and North Africa) and generally feeds on avian, small mammal, ungulate, and human hosts [16]. This tick species is a known vector of several pathogens of human health concern in the same genera as native USA pathogens including *Babesia* spp., *Brucella* spp., *Rickettsia* spp., *Theileria* spp. and several viruses (Tick-borne encephalitis, Crimean-Congo hemorrhagic fever, etc.) [17]. Furthermore, the red sheep tick has been expanding in its native range, exposing more humans to the pathogens it transmits [18].

The spread of endemic vectors has resulted in an increase in the incidence of human tick-borne diseases, which comprise over 90% of all reportable vector-borne diseases in the USA [2]. In recent years, certain native tick species have expanded their geographic distribution such as *A. americanum*, the lone star tick [3, 19], and *Haemaphysalis leporispalustris*, the rabbit tick [20]. Lone star ticks transmit several pathogens of human and veterinary concern such as ehrlichiosis, tularemia, and Heartland viruses and have been associated with the red meat allergy and Southern tick-associated rash illness (STARI), which produces rashes similar to Lyme disease [19]. While rabbit ticks do not commonly feed on humans, they are known vectors of *R. rickettsii* (agent of Rocky Mountain spotted fever), *Coxiella burnetii* (an agent of Q fever), and *Francisella tularensis* (causative agent of tularemia) [21]. These native vector range expansions are cause for public and veterinary health concern because of the potential pathogen expansion into new regions.

Here, we report the first known invasion and establishment of *H. punctata* in the western hemisphere and in the USA and the discovery of *H. longicornis* in the state of Rhode Island. Additionally, we report on the geographic range expansion of native tick species *A. americanum* and *H. leporispalustris* to a new region in the USA. Block Island, RI, is an island approximately 14 km south of the mainland, consisting of a 25.2 km^2^ land mass and is dominated by *Ixodes scapularis*, the blacklegged tick, native and migratory bird species, and a low diversity of mammals (white-footed mice, the Block Island meadow vole and white-tailed deer). Few residents remain on the island year-round (*n* < 1000), but the population drastically increases in the summer months from tourists and seasonal residents (*n* ≈12,000) [22]. The host composition and high annual influx of humans make Block Island an ideal location for studying vector invasion events, pathogen prevalence and diversity, and public health risk of tick-borne pathogens.

## Methods

### Environmental sampling and study sites

Ticks were collected throughout Block Island, RI, during the summer months (May–August) from 2010 to 2020. From 2010 to 2013, 105 residential properties were sampled for ticks by dragging a 1 m^2^ corduroy cloth along the property edge between the lawn and dense vegetation stopping every 10 m to remove attached ticks [22]. From 2014 to 2020 permanent grids were established at three locations: BI-1: 41°12′38.7"N, 71°34′21.4"W; BI-2: 41°09′47.6"N, 71°33′58.1"W; and BI-3: 41°09′25.2"N, 71°35′22.9"W. Flags were placed every 10 m at each grid node and grid size varied among the locations based on habitat suitability: BI-1 consisted of 15 × 4 nodes, BI-2 consisted of 10 × 6 nodes, and BI-3 consisted of 12 × 10 nodes. Biweekly small mammal trapping and tick dragging occurred at each of these grid sites annually. Three transect sites were established at Clayhead trail (CH: 41°12′32.2"N 71°33′48.2"W), Boy Scout campground (BS: 41°10′00.5"N 71°34′18.4"W) and the Maze trail (MZ: 41°12′59.2"N 71°33′39.8"W). Eight 100-m transects were dragged at each of these transect sites biweekly each season. Due to a global pandemic, drag sampling in 2020 only occurred for 1 day in early June at each of the grid sites and CH; no small mammal or avian sampling occurred in 2020.

Ticks were collected by drag sampling over natural deciduous forest and other vegetation throughout the established grids and along transects on the sides of hiking trails. The dominant vegetation at the field sites on Block Island was characterized by trees [shad (*Amelanchier canadensis*), chokecherry (*Prunus virginiana*), black cherry (*Prunus serotina*)] and tall shrubs and brush [arrowwood (*Viburnum dentatum*), bayberry (*Myrica pensylvanica*), blackberry (*Rubus* spp.), multiflora rose (*Rosa multiflora*), and Japanese barberry (*Berberis thunbergii*)]. The average temperature on Block Island during our sampling season (May–August) was 22.1 °C high (range 16.7–25 °C) and 14.9 °C low (range 9.4–17.8 °C) with an average precipitation of 7.5 cm (range 6.7–8.6 cm) (data calculated from the USA Climate Data website specifically for Block Island).

### Wildlife host sampling

Small mammals (mostly *Peromyscus leucopus* and *Microtus pennsylvanicus provectus*) were sampled for ticks using Sherman traps baited with peanut butter, oats, and sunflower seeds at each of the grid locations. Avian species were sampled at three locations on Block Island (North Island, NI: 41°12′43.3"N 71°33′48.7"W; Lapham, LP: 41°12′34.9"N 71°33′49.9"W; Ocean View Pavilion, OVP: 41°10′17.7"N 71°33′18.4"W) using mist netting techniques.

### Tick species identification

*Haemaphysalis* spp. ticks were identified morphologically (confirmed by the National Veterinary Services Laboratory-NVSL) and molecularly using cytochrome oxidase 1 (*cox1*) primers [23]. *Amblyomma americanum* ticks were morphologically identified to species using specific keys [24].

## Results and discussion

### The red sheep tick

**Fig. 1:**
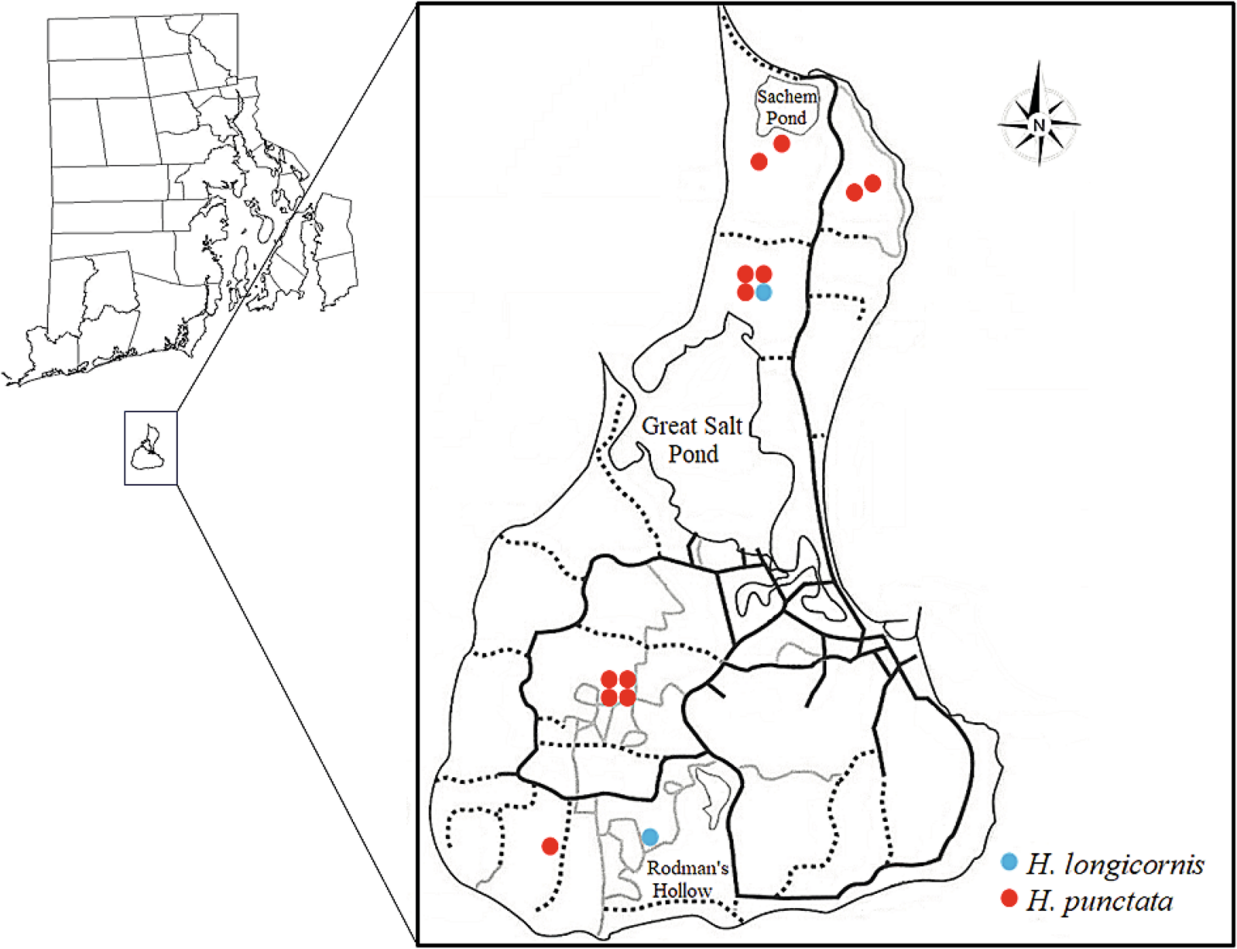
Map of Block Island showing the locations where invasive species *Haemaphysalis punctata* (red circles) and *H. longicornis* (blue circles) were collected; each circle represents a single individual

Two adult female *H. punctata* were first collected from northern Block Island locations in May 2010 (Fig. 1) and this species was observed in low abundance each year until 2020 (Table 1). This is the first documented discovery of the red sheep tick collected from the environment in the western hemisphere and the USA. Sequences of *H. punctata* were confirmed via GenBank using BLASTn (NCBI) and most closely identified with accession numbers JX394186 and JX394187 (identity score ≥ 91.17%; query coverage ≥ 85%). A few *H. punctata* potential invasion events have been reported over the years; for instance, in 1989, an adult female was collected from ostriches imported from Portugal during USA quarantine [25]. Additionally, in 2006, the NVSL identified an adult male *H. punctata* collected by the Animal and Plant Health Inspection Service (APHIS) Plant Protection and Quarantine inspectors from a trophy animal hide (European mouflon, *Ovis gmelini*) arriving from the Ukraine at O’Hare International Airport. Most recently, in June 2019, NVSL identified a female *H. punctata* collected at the APHIS New York Animal Import Center in Orange County, New York, from a horse imported from The Netherlands (pers. comm. James Mertins). To date, both adults (males and females) and larvae have been recovered at our study sites, suggesting that this species is reproducing, overwintering, and feeding on hosts in the environment. Because this species has been recovered almost every year for the last 10 years, greater than six individuals of one life stage, and different life stages have been recovered, this species can be considered established on Block Island [26].

**Table 1:** The number of each tick species collected from each year on Block Island

Year	*Amblyomma americanum*			*Haemaphysalis leporispalustris*			*Haemaphysalis longicornis*			*Haemaphysalis punctata*		
	A	N	L	A	N	L	A	N	L	A	N	L
2010	1	–	–	–	3	–	–	–	–	2	–	–
2011	–	–	–	–	–	–	–	–	–	–	–	–
2012	–	1	–	–	–	–	–	–	–	1	–	–
2013	1	1	–	–	–	–	–	–	–	1	–	2^a^
2014	–	–	–	–	–	–	–	–	–	–	–	–
2015	1	1	–	–	–	–	–	–	–	–	–	–
2016	–	9	–	–	–	–	–	–	–	–	–	2
2017	5	4	–	–	1	–	–	–	–	1	–	–
2018	2	41	1	–	1	2^a^	–	–	1	2	–	–
2019	4	4	1322	–	–	–	1	–	–	1	–	–
2020^b^	4	46	–	–	–	–	–	–	–	1	–	–
Total	18	107	1323	0	5	2	1	0	1	9	0	4

^a^Denotes the number of avian-derived ticks

^b^Ticks were only collected via dragging 1 day in early June

*A* adult, *N* nymph, *L* larvae

The red sheep tick is native to the Palearctic region, but their country of origin and the method of introduction into the USA are not yet clear. Immature-stage ticks may have arrived attached to migratory birds as some European birds have been documented on Block Island (pers. comm. Kimberley Gaffett and Rachel Farrell). No *H. punctata* were found on small mammals. While ungulates and medium-sized mammals are the most common hosts of *H. punctata* in their native range, two partially engorged larvae were removed from a common yellowthroat (*Geothlypis trichas*) in 2013, providing evidence that this species does occasionally feed on avian hosts and is feeding on native species. Tourists along with their pets and seasonal workers from around the globe annually visit the island and may have inadvertently introduced this tick to Block Island. In its native range, *H. punctata* is commonly reported feeding on humans [17], increasing the possibility that this species may be a significant threat to human health. Further investigation of the pathogens this species harbors and may be able to transmit to humans and wildlife is currently underway. Importation of livestock and nursery stock from the native range of *H. punctata* is not common on Block Island. This tick species is environmentally adaptable to a wide range of climatic conditions in its native range, which would allow it to successfully establish on Block Island and potentially the New England area.

### The Asian longhorned tick

A single larval *H. longicornis* was collected in 2018 from BI-1 in the northern part of the island; the following year an adult was collected from BI-3 in the south (Table 1). This constitutes the first report of this species in Rhode Island (Fig. 1). Sequences were compared to those available on GenBank (BLASTn, NCBI), and accession number MK439888 was the closest match (identity score ≥ 98.53%; query coverage ≥ 98%). The ability of this species to reproduce parthenogenetically and the large population of white-tailed deer, a preferred host of *H. longicornis* [14, 27, 28], may facilitate the spread of this invasive tick on Block Island. Very few pathogens have been recovered from *H. longicornis* in some invaded regions of the USA [29], and their ability to vector native pathogens is still under investigation [11, 12].

### The Lone star tick

While *A. americanum* are native species in the southern and eastern parts of the USA [19], Block Island is a newly invaded and established region for this species. From 2010 to 2017, very few *A. americanum* ticks were recovered each year (*n* < 10). All three life stages and an increase in the number of ticks were first observed at one site in 2018, and the population greatly increased in 2019 (Table 1). Sampling in 2020 was restricted to only early June and was too limited to compare with previous years. The lone star tick has been steadily increasing and spreading to new locations across the island (Additional file 1: Table S1) and may eventually displace or replace the blacklegged tick as the dominant species in this area [30]. This increase in abundance of *A. americanum* may result in new pathogens spreading across the island that may be of concern for residents and tourists. Pathogen screening and continued surveillance of *A. americanum* across the island will be important to better assess the potential human health risks this species may pose.

### The rabbit tick

*Haemaphysalis leporispalustris* has a wide distribution throughout the Americas. However, it is interesting to note its presence in an environment devoid of its preferred host species, lagomorphs or other medium-sized mammals. It is suspected that immature ticks arrived and survive on ground-feeding avian hosts as no adults have been found to date. This hypothesis is further supported by the fact that two partially engorged larvae were recovered from a Connecticut warbler (*Oporornis agilis*) in 2018 in the northern part of the island (Table 1).

Block Island is characterized by a depauperate mammalian host community; therefore, it was surprising to find three different species of *Haemaphysalis* congregating on the same small island. Moreover, the tick species reported are not found in small mammals but rather on birds and ungulates, calling attention to additional surveillance of these hosts.

## Conclusions

This is the first report of *H. punctata* in the western hemisphere, the first report of *H. longicornis* in Rhode Island, and the first occurrence of *A. americanum* and *H. leporispalustris* on Block Island. Pathogen prevalence and the number of different pathogens in the environment on Block Island may increase given the potential introduction of exotic pathogens and the close confined proximity of wildlife hosts and humans. The introduction, establishment, and subsequent increase in *A. americanum* population size across the island may result in new pathogens on the island, the potential displacement or replacement of *I. scapularis,* and an increase in human risk for different pathogens. White-tailed deer and ground-nesting avian species are the most likely hosts of the invasive and native species discussed on Block Island as none of these tick species were recovered from small mammals. Screening for a wide variety of pathogens and continued surveillance of these invasive and native tick species are crucial for determining the risk they may pose to human and wildlife health.

## Supplementary Information

**Additional file 1: Text S1.***Amblyomma americanum* distribution on Block Island by year and collection site.

## Data Availability

The data sets used and analyzed in the present study are included in this article.

## Abbreviations

PCR: Polymerase Chain Reaction
RI: Rhode Island
BI: Block Island
CH: Clayhead trail
BS: Boy Scout campground
MZ: Maze trail
NI: North Island
LP: Lapham
OVP: Ocean View Pavilion
NVSL: National Veterinary Services Laboratory
NCBI: National Center for Biotechnology Information
BLASTn: Basic Local Alignment Search Tool nucleotide
APHIS: Animal and Plant Health Inspection Service
