# Supplementary material for: First hemispheric report of invasive tick species Haemaphysalis punctata, first state report of Haemaphysalis longicornis, and range expansion of native tick species in Rhode Island, USA
Source: Parasit Vectors. 2021 Aug 10;14:394. doi: 10.1186/s13071-021-04887-z (PMC8353422; doi:10.1186/s13071-021-04887-z)
Supplement: Supplementary file 1 — Additional file 1: Text S1.Amblyomma americanum distribution on Block Island by year and collection site. [file 13071_2021_4887_MOESM1_ESM.docx]

**Additional file 1: Table S1.** *Amblyomma americanum* distribution on Block Island by year and collection site.

| Year | Site | Life stage | | |
| --- | --- | --- | --- | --- |
|  |  | **A** | **N** | **L** |
| 2010 | NRP | 1 | 0 | 0 |
| 2012 | MZ | 0 | 1 | 0 |
| 2013 | BI-3 | 1 | 1 | 0 |
| 2015 | BI-3 | 1 | 1 | 0 |
| 2016 | BI-1 | 0 | 7 | 0 |
|  | BI-2 | 0 | 1 | 0 |
|  | MZ | 0 | 1 | 0 |
| 2017 | BI-3 | 4 | 4 | 0 |
|  | MZ | 1 | 0 | 0 |
| 2018 | BI-1 | 1 | 26 | 0 |
|  | BI-3 | 1 | 12 | 0 |
|  | MZ | 0 | 3 | 1 |
| 2019 | BI-1 | 1 | 0 | 547 |
|  | BI-2 | 1 | 1 | 2 |
|  | BI-3 | 0 | 1 | 15 |
|  | CH | 1 | 1 | 1 |
|  | MZ | 1 | 1 | 757 |
| 2020^a^ | BI-1 | 4 | 27 | 0 |
|  | BI-2 | 0 | 1 | 0 |
|  | BI-3 | 0 | 17 | 0 |
|  | CH | 0 | 1 | 0 |
| Total |  | 18 | 107 | 1323 |

^a^Collection only occurred one day in early June and only at BI-1, BI-2, BI-3, and CH.

*Abbreviations*: A, adult; N, nymph; L, larvae; BI-1, Block Island grid site 1; BI-2, Block Island grid site 2; BI-3, Block Island grid site 3; CH, Clayhead trail; MZ, Maze trail; NRP, Northern residential property.
